# The discriminatory value of cardiorespiratory interactions in distinguishing awake from anaesthetised states: a randomised observational study

**DOI:** 10.1111/anae.13208

**Published:** 2015-09-09

**Authors:** D. A. Kenwright, A. Bernjak, T. Draegni, S. Dzeroski, M. Entwistle, M. Horvat, P. Kvandal, S. A. Landsverk, P. V. E. McClintock, B. Musizza, J. Petrovčič, J. Raeder, L. W. Sheppard, A. F. Smith, T. Stankovski, A. Stefanovska

**Affiliations:** ^1^ Lancaster UniversityLancasterUK; ^2^ Oslo University HospitalUllevaalNorway; ^3^ Jožef Stefan InstituteLjubljanaSlovenia; ^4^ Royal Lancaster InfirmaryLancasterUK; ^5^ Faculty of Mathematics and PhysicsUniversity of LjubljanaLjubljanaSlovenia

## Abstract

Depth of anaesthesia monitors usually analyse cerebral function with or without other physiological signals; non‐invasive monitoring of the measured cardiorespiratory signals alone would offer a simple, practical alternative. We aimed to investigate whether such signals, analysed with novel, non‐linear dynamic methods, would distinguish between the awake and anaesthetised states. We recorded ECG, respiration, skin temperature, pulse and skin conductivity before and during general anaesthesia in 27 subjects in good cardiovascular health, randomly allocated to receive propofol or sevoflurane. Mean values, variability and dynamic interactions were determined. Respiratory rate (p* *=* *0.0002), skin conductivity (p* *=* *0.03) and skin temperature (p* *=* *0.00006) changed with sevoflurane, and skin temperature (p* *=* *0.0005) with propofol. Pulse transit time increased by 17% with sevoflurane (p* *=* *0.02) and 11% with propofol (p* *=* *0.007). Sevoflurane reduced the wavelet energy of heart (p* *=* *0.0004) and respiratory (p* *=* *0.02) rate variability at all frequencies, whereas propofol decreased only the heart rate variability below 0.021 Hz (p* *<* *0.05). The phase coherence was reduced by both agents at frequencies below 0.145 Hz (p* *<* *0.05), whereas the cardiorespiratory synchronisation time was increased (p* *<* *0.05). A classification analysis based on an optimal set of discriminatory parameters distinguished with 95% success between the awake and anaesthetised states. We suggest that these results can contribute to the design of new monitors of anaesthetic depth based on cardiovascular signals alone.

## Introduction

Because general anaesthesia involves loss of consciousness, objective measures of depth of anaesthesia have been focused mostly on EEG and EEG‐derived measurements, such as the evoked potentials from sound or noxious stimuli 1, 2, 3, 4. Currently, there is no monitor of brain activity which meets the ideal of 100% sensitivity and specificity for the awake and anaesthetised states. At least part of that deficiency may arise because ‘anaesthesia vs awake’ may not be a binary set of conditions, and there may be neurophysiological states in between that are nonetheless apparently suitable for surgery 5, 6. However, anaesthesia is also well known to influence the cardiovascular system. A multi‐centre European study recently assessed a combination of EEG parameters with measures of standard clinical cardiovascular parameters (heart rate, blood pressure, change of heart rate and blood pressure), for characterisation of the anaesthetic state 7. Even with this extensive combination of measures, the prediction probability of 88% for correctly identifying the awake‐to‐anaesthetised transition still fell far below the 100% ideal.

In standard clinical settings, like the above study, most cardiovascular data are processed on a beat‐by‐beat basis. But, if measured with a higher sampling frequency (i.e. several samples per beat), the resultant signals can provide more information relating to how the system is changing (its dynamic properties such as contributing oscillations and their coherence). In turn, how these change with anaesthesia can be assessed. There is also the possibility of including additional non‐invasive sensors, such as respiratory effort, skin conductivity and skin temperature, all operated at a high sampling frequency. We therefore propose that simultaneous measurements of cardiovascular signals in combination with dynamic analysis could have potential clinical applications by providing the basis for future depth of anaesthesia monitors.

As all cardiovascular variables vary with time, and do so in a non‐linear way, analyses that reflect this non‐linearity provide closer approximations to reality 8, 9. Dynamic analysis of blood flow measurements suggests that there are at least five characteristic oscillatory frequencies in the cardiovascular system, each attributable to a particular physiological process, including cardiac, respiratory, myogenic, sympathetic and endothelial activities (Table 1). Complex oscillations can often be decomposed into their individual oscillatory components using the Fourier transform. Formally, however, this can be done only when the signals are periodic, which implies that, for example, mean heart rate is constant over time. In reality, this is not the case. Therefore, we need methods that reveal the time‐variability of the constituent oscillations. A method known as ‘wavelet analysis’ can decompose oscillatory components locally in time to yield an optimal time‐frequency resolution 8, 10. Furthermore, techniques such as ‘wavelet phase coherence’ and ‘synchronisation analysis’ can extract information about the interactions between the underlying oscillatory processes 9, 11.

**Table 1:** Frequency intervals and their associated physiological activity

Interval	Frequency (Hz)	Physiological origin
I	0.6–2.0	Heartbeat
II	0.145–0.6	Respiratory activity
III	0.052–0.145	Intrinsic myogenic activity
IV	0.021–0.052	Neurogenic (sympathetic) activity
V	0.0095–0.021	NO‐dependent endothelial activity

NO, nitric oxide.

We expected that anaesthesia would result in multiple changes in cardiovascular regulation that could be revealed by applying novel non‐linear dynamics methods to relevant signals. In addition, we proposed that, when a large range of cardiovascular and autonomic parameters are analysed, it would be possible to reliably establish whether a patient was awake or anaesthetised. Thus, we tested two hypotheses: first, that there is a clear difference between the awake and anaesthetised states; and second, that anaesthesia using propofol would be demonstrably different (as assessed by our methods) from anaesthesia using sevoflurane.

The choices of signals to be recorded, and the best ways of creating the parameters for their interaction, were guided by the results of earlier work on cardiovascular dynamics during anaesthesia in both rats 12, 13 and humans 14. Core temperature 15 and skin conductance 16 were added to the signals to be recorded because these quantities are known to undergo changes during anaesthesia.

## Methods

The approvals of the relevant Research Ethics Committees were obtained before the study commenced. Data collection took place before registration of studies into trials registries was required. Subjects were invited to participate and their written informed consent to take part in the study was obtained on the day of surgery. We studied healthy (ASA physical status 1 and 2) subjects aged between 18 and 60 years scheduled to undergo minor to intermediate surgical procedures. The exclusion criteria were: cardiovascular disease or chronic obstructive pulmonary disease; ongoing cancer or diabetes mellitus; allergy to anaesthetic drugs; or taking medication that could influence the central nervous system or cardiovascular dynamics. Subjects were ineligible if they had consumed caffeine‐containing drinks after 20:00 the previous day, or had taken a sleeping pill the night before surgery.

A randomisation envelope was opened by study personnel and the specified anaesthetic drug was prepared, checked and labelled. No premedication was given. Standard clinical anaesthetic monitoring was attached (pulse oximetry, 3‐lead electrocardiogram and non‐invasive arterial blood pressure). The sensors for data recording were then applied as described below. Intravenous access was obtained. After application and calibration of the sensors, a stabilisation period was allowed.

Once subjects confirmed that they were comfortable, they were asked to relax, lie still, stay awake, and not speak unless necessary for the duration of the recordings. The first set of recordings was then made for approximately 30 minutes before anaesthesia. After this, anaesthesia was induced with the drug dictated by the randomisation procedure. In addition, a commercial gas analyser displaying respired gas oxygen, carbon dioxide and volatile anaesthetic agent concentrations was used. For the propofol group, anaesthesia was induced by infusing propofol until a simulated plasma target concentration of 6.0 μg.ml^−1^ was reached (Diprifusor^™^, target control system (TCI) Astra‐Zeneca, London, UK). A laryngeal mask airway was inserted 2 min after the start of the infusion. After insertion, the target concentration was reduced to 3.0 μg.ml^−1^ and the infusion was maintained at this rate throughout the measurement period 17, 18. For the sevoflurane group, subjects were asked to breathe sevoflurane through a close‐fitting facemask until an end‐tidal concentration of 5% was reached. A laryngeal mask airway was inserted, and then the sevoflurane turned off until the end‐tidal concentration fell to 2%. The sevoflurane was then re‐instituted to maintain the end‐tidal concentration at 2% throughout the measurement period 19. After a further stabilisation period of 5–10 min, the second set of signal recordings took place. Subjects breathed spontaneously during both sets of recordings.

The signals were recorded using a system (Cardio&Brain Signals; Jožef Stefan Institute, Ljubljana, Slovenia) specially designed for this study. The signals were fed, via 24‐bit A/D conversion at 1200 Hz, into a purpose‐built signal‐conditioning unit and then stored on a laptop computer.

The electrical activity of the heart was measured with a 3‐lead ECG system. To obtain well‐defined ECG R‐peaks, used to calculate heartbeat timing, the standard electrodes were attached to the subject's left shoulder, right shoulder and lowest rib on the left side of the body. The connecting cable was a M1735A 3‐lead ECG shielded cable (Philips Medizin Systeme Böblingen GmbH, Boeblingen, Germany) and adapted to the Cardio&Brain Signals ECG input connector.

Respiratory effort was recorded using a belt encircling the subject's chest, fitted with a Biopac TSD201 Respiratory Effort Transducer (Biopac Systems Inc., Goleta, CA, USA).

Skin temperature was measured with two 8.5‐mm diameter, high‐sensitivity, low heat capacity thermistors taped to the skin: YSI 709B Thermilinear^®^ sensors (YSI Inc., Yellow Springs, OH, USA). The first (T1) was positioned on the inside right ankle, over the medial malleolus; and the second (T2) on the inside of the right wrist, over the radial styloid process. Care was taken to ensure good thermal contact between the sensor and the skin.

Skin conductivity was measured using a pair of silver‐plated electrodes taped to the ball and between the distal and proximal joints of the right thumb. The electrodes were adapted from the M1931A Reusable EEG Adult Cup Electrode set of the Ag/AgCl electrode system (Philips Medizin Systeme Böblingen GmbH). Electrical contact was facilitated by use of a standard conductivity gel, e.g. Electro‐Gel^®^ from Electro‐Cap International, Inc., Eaton, OH, USA. Conductivity was determined from the DC current when 0.5 V was applied between the electrodes. The maximum current was limited to 125 μA.

The pulse generated by changes in arterial blood pressure was measured on the subject's right index finger with a piezoelectric pressure transducer, the MLT1010 Pulse Transducer (AD Instruments Pty Ltd, Bella Vista, NSW, Australia). The volume of blood flowing into the finger tightened a cuff, generating a pulse signal. As the output is a measure of dynamic changes in pressure, the signal was integrated to obtain a time series of blood pressure.

The R‐peaks of the ECG signal, and the maxima of the respiration signal, were used as markers for cardiac and respiratory oscillations, respectively. In this way, the heart rate variability and respiratory frequency variability signals were obtained (Appendix S1). The maxima and minima of the integrated pulse signal represent the systolic and diastolic pressures, respectively. We defined the arrival times of the pulse signal to the finger as the minima in the pressure; this is not only the easiest part of the signal to recognise and locate but is also the least distorted part of the propagated wave 20. The time delay between the arrival time of the pressure pulse wave and its corresponding R‐peak was defined as the pulse transit time.

To discern the frequency content of the cardiovascular oscillations, we performed wavelet analysis on the recorded signals. The Morlet mother wavelet 21 was used to calculate the continuous wavelet transform. The spectral range was then divided into the five physiologically relevant frequency intervals 8, 22 between 0.0095 and 2 Hz shown in Table 1. The energy of each of the signals, including the variability of the heart and respiration rates, was calculated (Appendix S2) within these intervals.

Wavelet phase coherence was used for exploration of the phase relationships at particular frequencies between oscillations from pairs of separate signals 23, 24. Generally, phase coherence was considered to exist if the phase difference between two oscillations remained constant throughout the whole time of observation; this suggested that the signals are regulated from a common source, or that they are mutually synchronised 25 (see Appendix S2 for details of how the synchronisation and wavelet phase coherence were calculated; the method of surrogates 26 was used to test for significance). Co‐ordination between the heart and respiration is known to exist at rest and during anaesthesia 9, 11, 12, 27, 28, 29, 30, 31. Specifically, we studied the phase synchronisation between respiration and heartbeat as analysed by the synchronisation index 8. The synchronisation time for each subject was defined as the sum of the intervals during which the indices are 95% or above of perfect synchronisation for window lengths of 6T for 1:n synchronisation and 8T for 2:n synchronisation, where T is the average respiratory period. This choice of window length and the use of phase gives the same relative period for all subjects, as opposed to methods which are based on absolute time 30, 31. We then calculated the change in synchronisation time between the awake state and during anaesthesia.

The analyses were made in MATLAB (MathWorks, Natick, MA, USA) and two types of comparison were carried out. First, using a paired, non‐parametric (Wilcoxon signed‐rank) test, we investigated the statistical differences between the extracted parameters related to the awake and anaesthetised states. Secondly, we tested statistical differences between the extracted parameters relevant for the anaesthetised state with each of the two agents, using an unpaired Wilcoxon rank sum test. The significance level was set at p* *=* *0.05. Wherever appropriate, the significance of dynamic parameters, like phase coherence, was tested against properties extracted from surrogate signals which were generated by randomising the correlations between the two signals, thus making them independent and phase incoherent, but preserving the statistical properties of each signal 26.

On the basis of parameters derived from the recorded signals, and by applying automatic classification method, we classified subjects into *groups* or *classes* associated with three distinct states: awake, anaesthetised with sevoflurane, and anaesthetised with propofol. The classification is a machine‐learning process which is refined until it performs optimally. There are many possible classification schemata and training methods 32, 33, 34, 35. We applied two different approaches. First, we applied a method specially developed for this study. It is a variant of distance‐based classification (see below) that arose naturally in the present context. It takes direct account of the physiological nature of the extracted parameters and during classification simultaneously optimises the distance measure. Secondly, to test the strength of our new classification method, we applied standard classification techniques and corresponding methodology from the freely available open‐source software package WEKA 36.

We used an unpaired test (Kolmogorov–Smirnov 37) to determine whether there were significant differences between the classified groups. If the difference for a pair of physiological variables was found to be significant (p* *<* *0.05) these were considered as attributes and were included in the discriminatory analysis. The attributes, summarised in Table 2, were grouped into mean values, wavelet powers and interactions; we then went through a controlled cascade of reductions and mergers of attribute sets to find the most predictive attributes (see online Appendix S3 for details on ranking attributes).

**Table 2:** The attributes used in the vector‐based discriminatory analysis for the different subsets of data. Roman numerals indicate frequency intervals (see Table 1). The 12 most relevant attributes, used in the optimised classification calculation, appear shaded

Mean values subset	Wavelet powers subset	Interactions subset
Heart rate	HRV energy II	HRV‐conductivity II
Respiratory rate	HRV energy III	HRV‐conductivity III
Skin temperature	HRV energy IV	HRV‐conductivity IV
Skin conductivity	HRV energy V	Conductivity‐pulse I
Pulse transit time	RFV energy III	Conductivity‐pulse III
Total HRV energy	RFV energy IV	Conductivity‐pulse IV
Total RFV energy	RFV energy V	Conductivity‐temperature I
Total conductivity energy	Conductivity energy III	Conductivity‐temperature II
Total temperature energy	Conductivity energy IV	Pulse‐temperature I
	Conductivity energy V	C‐R synchronisation time
	Conductivity energy VI	1:n synchronisation window length
	Temperature energy III	2:n synchronisation window length
	Temperature energy IV	
	Temperature energy V	
	Temperature energy VI	

RFV, respiratory frequency variability; HFV, heart rate variability.

We used a distance‐based classification technique, called the nearest neighbour classifier 31, where each subject is assigned a vector x based on their measured signals and derived values. The (squared) distance between two subjects x_1_ and x_2_ is calculated as D(x_1_,x_2_)^2^ = (x_1_ − x_2_)^T^ A (x_1_ − x_2_)*,* where T indicates the transpose of the original matrix. The distance measure (i.e. the entries in the positive symmetric matrix A) is determined through a procedure described in online Appendix S3, where further details are given [38].

The results of a classifier are assessed by inspecting the ‘confusion matrix’ [38] (see Table A2 of the online supplementary information). In brief, the confusion matrix contrasts actual states/classifications (rows) with classifications/states (columns) predicted by the classifier. The diagonal entries correspond to the number (likelihood) of correct classifications and off‐diagonal entries to the number (likelihood) of misclassifications: the likelihood entries in one row sum up to 100%. Further details can be found in Appendix S3.

## Results

We recruited 27 patients from two centres, Oslo (n = 13) and Lancaster (n = 14). Of these, 12 were anaesthetised with propofol and 15 with sevoflurane; six patients received propofol in each of the two centres; eight received sevoflurane in Lancaster and seven in Oslo. The subject characteristics are given in Table 3.

**Table 3:** Characteristics of the two groups. Values are number or mean (SD)

Group	Number of subjects (M:F)	Age; years	Height; cm	Weight; kg	Body mass index; kg.m^−2^
Sevoflurane	15 (8:7)	31.9 (9.4)	176.0 (11.5)	77.8 (12.3)	25.7 (5.0)
Propofol	12 (10:2)	38.5 (12.3)	178.9 (9.1)	78.6 (16.3)	24.0 (3.9)

**Figure 1:**
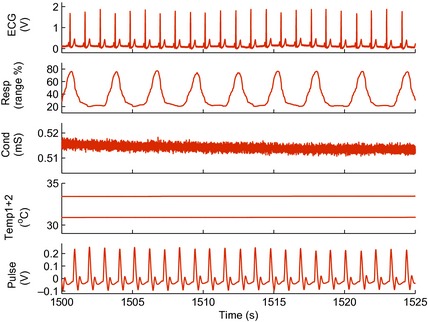
Example of a short segment of signals recorded during anaesthesia (from top to bottom): electrical activity of the heart (ECG); respiration as a percentage of the sensor range; skin conductivity; skin temperature from the wrist (upper) and ankle (lower) and piezoelectric pulse.

An example of a set of recorded signals is given in Fig. 1. We first calculated their mean values across the whole recording, as shown in Table 4. Each subject acted as his/her own control in terms of awake versus anaesthetised. There was no statistically significant difference between the awake measurements of the two study groups, propofol and sevoflurane.

**Table 4:** Heart rate, respiratory rate, skin temperature, skin conductivity, pulse transit time, the mean wavelet energy of the signals, the duration of cardiorespiratory synchronisation and the duration of the windows for 1:n and 2:n cardiorespiratory synchronisation. Values are mean (SD)

	Sevoflurane			Propofol			Comparison	
	Control	Anaes	p	Control	Anaes	p	p_controls_	p_sevo.prop_
HR; Hz	1.07 (0.16)	1.07 (0.15)	0.89	1.08 (0.18)	1.14 (0.20)	0.23	0.96	0.46
RR; Hz	0.22 (0.07)	0.37 (0.08)	0.0002	0.19 (0.05)	0.23 (0.05)	0.09	0.14	0.0002
STemp; °C	30.0 (0.9)	32.1 (1.2)	0.00006	30.2 (2.1)	31.9 (2.2)	0.0005	0.33	0.75
SCond; ms	2.09 (2.50)	1.34 (1.17)	0.03	2.26 (2.18)	1.67 (1.37)	0.18	0.69	0.29
PTT; s	0.18 (0.02)	0.21 (0.05)	0.02	0.18 (0.01)	0.2 (0.02)	0.007	0.17	0.68
Mean wavelet energy								
HRV; Hz^2^	1.36	0.53	0.0004	0.98	0.47	0.11	0.07	0.98
RFV; Hz^2^	0.26	0.13	0.02	0.39	0.14	0.051	0.54	0.21
STemp; °C^2^	0.00002	0.00007	0.14	0.0002	0.0001	0.85	0.07	0.54
SCond; S^2^	0.09	0.0003	0.002	0.02	0.0003	0.003	0.75	0.61
C‐R synch.; s	173	385	0.03	138	323	0.04	0.55	0.55
C‐R 1:n synch. window; s	38.00	16.00	0.00006	36.41	27.67	0.0830	0.0596	0.000022
C‐R 2:n synch. window; s	50.87	21.20	0.00006	48.50	36.83	0.0654	0.0824	0.000019

p values are provided for the comparisons between control and anaesthetised groups with each of the two anaesthetics, and in the final columns between the awake measurements for the two groups (p_controls_) and for the two anaesthetic agents (p_sevo.prop_).

HR, heart rate; RR, respiratory rate; STemp, skin temperature; SCond, skin conductivity; PTT, pulse transit time; C‐R synch, duration of cardiorespiratory synchronisation.

Neither anaesthetic agent caused a statistically significant change in the mean heart rate and there was no significant difference between the anaesthesia groups; p values for all comparisons are shown in Table 4. In contrast, sevoflurane caused a significant increase in respiratory rate compared both with baseline and with propofol.

A significant increase in skin temperature was observed on both sites with both anaesthetic agents. The mean values shown in Table 4 represent the mean between the two measurement sites, on wrist and ankle. There was no significant difference between the propofol and sevoflurane groups.

There was wide between‐subject variation in recorded skin conductivity, amounting to several orders of magnitude. However, for all subjects, skin conductivity was typically highest when the sensor was first attached, then fell during the period of awake measurement, with rapid increases when the subject was subjected to a stimulus such as noise, end of the measurement, or the onset of the anaesthesia procedure. Mean skin conductivity decreased during anaesthesia with both agents, though this was significant only for sevoflurane. There was no significant difference between the two agents with respect to this decrease.

Anaesthesia caused a significant increase in pulse transit time (Table 4): 17% with sevoflurane and 11% with propofol.

**Figure 2:**
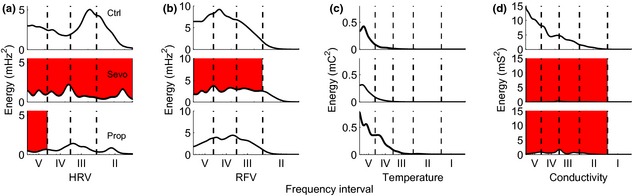
Time‐averaged energy in the wavelet transform (mean values from all subjects), for (a) heart rate variability, (b) respiratory frequency variability, (c) skin temperature and (d) skin conductivity. The top row is the awake control (Ctrl), with sevoflurane (Sevo) and propofol (Prop) underneath. Red regions denote where there is a statistically significant difference (p* *<* *0.05) between the control and anaesthetised state. I to V are frequency intervals as indicated in Table 1.

Wavelet analysis of oscillations in measured and derived signals revealed the following results. Both anaesthetic agents were associated with a decrease in heart rate variability (Fig. 2); this was significant across all frequencies for sevoflurane, but only in frequency interval V (0.0095–0.021 Hz) for propofol. Both agents were associated with a decrease in respiratory frequency variability, significantly for sevoflurane and close to significance for propofol. There was no significant change in temperature variability for either agent. However, median skin conductivity variability decreased considerably for both agents, though this fall was small in comparison with the between‐subject variation in mean values at baseline.

Furthermore, we observed that the heartbeat and breathing entered periods of synchronisation, whereby a fixed number of beats occurred for each breathing cycle, as had been demonstrated previously 27, 28, 29, 30, 31. The total synchronisation time was found to increase with both anaesthetics, whereas the 1:n and 2:n synchronisation windows decreased significantly only with sevoflurane, thus showing significant difference in the effects of the two anaesthetics. In contrast, the difference in the synchronisation time between the two anaesthetics was not significant (Table 4).

**Figure 3:**
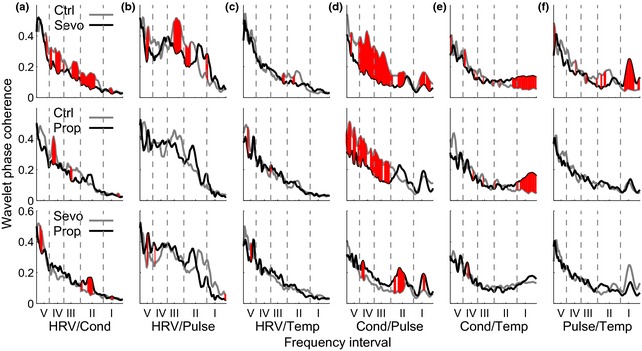
Comparisons of wavelet phase coherence. Each curve is obtained as an average over all subjects in a particular group. Top row: controls (grey) compared with sevoflurane‐induced anaesthesia (black). Middle: controls (grey) and propofol‐induced anaesthesia (black). Bottom: the two anaesthetic agents compared with each other. In each case, the comparisons are shown for coherence between: (a) HRV and skin conductivity; (b) HRV and pulse; (c) HRV and skin temperature; (d) skin conductivity and pulse; (e) skin conductivity and skin temperature; and (f) pulse and skin temperature. Red‐shaded regions denote statistically significant differences (p* < *0.05) between the two plots at the frequencies indicated. I–V are frequency intervals as indicated in Table 6.

Figure 3 shows the phase coherence between pairs of the cardiovascular and respiratory signals in the awake and anaesthetised states. Frequencies where the change is significant are shaded; for example, section (d) of Fig. 3 shows the phase coherence between skin conductivity and the pulse pressure signal. Both sevoflurane and propofol caused a significant reduction in phase coherence below 0.145 Hz. In effect, the coherence between these two signals when awake was lost during anaesthesia.

The attributes used in the classification analysis to distinguish the awake from the anaesthetised state appear in Table 2. The controlled cascade of reductions and mergers of attribute sets revealed a very efficient set of 12 attributes, which appear shaded in Table 2 (see Appendix S3 online for details on ranking attributes). By using the obtained optimal distance in classification with repeated hold‐out validation, we obtained a classification accuracy of A_3_ = 90% with the corresponding confusion matrix expressed in percentages in Table 5. In this table, the sum in a horizontal row is 100% and the values on the diagonal present correct classifications. Thus, 98% of awake subjects were classified as awake and 2% as anaesthetised with propofol. The classification resulted in recognising 84% of subjects anaesthetised with sevoflurane correctly, whereas 8% were wrongly classified as awake and another 8% as anaesthetised with propofol. Lastly, of all subjects anaesthetised with propofol, 80% were classified correctly, 9% were classified as awake and 11% as anaesthetised with sevoflurane. Further description of the confusion matrix is given in Appendix S3.

**Table 5:** Confusion matrix, giving the likelihoods of correct and incorrect classification into three states for distance‐based classification using an optimal distance on 12 attributes

	Classified state		
	Awake	Anaes‐Sevoflurane	Anaes‐Propofol
Actual state			
Awake	98%	0	2%
Anaes‐Sevoflurane	8%	84%	8%
Anaes‐Propofol	9%	11%	80%

Merging the two anaesthetised states into one state, yielded a classification accuracy of A_2_ = 95%, as summarised in the confusion matrix given in Table 6. The classification with the optimal distance and simple leave‐one‐out cross‐validation gives an accuracy A_3_ = 98%, as summarised in the confusion matrix in Table 7.

**Table 6:** Confusion matrix, giving the likelihoods of correct and incorrect classification into two states, for the distance‐based classification as in Table 3, using an optimal distance on 12 attributes

	Classified state	
	Awake	Anaesthetised
Actual state		
Awake	98%	2%
Anaesthetised	8%	92%

**Table 7:** Confusion matrix giving both the numbers and likelihoods of correct and incorrect classifications into three states, for the distance‐based classification as in Table 3, using an optimal distance on 12 attributes. The numbers have been obtained using leave‐one‐out cross‐validation (as opposed to repeated 50% hold‐out for Tables 3 and 4)

	Classified state		
	Awake	Anaes‐Sevoflurane	Anaes‐Propofol
Actual state			
Awake	100%	0	0
Anaes‐Sevoflurane	7%	93%	0
Anaes‐Propofol	0	0	100%

Details of the classification analysis and results are summarised in Appendix 3.

## Discussion

The use of cardiovascular and autonomic signs to assess depth of anaesthesia is not new; scores based on such signs have been used in the research context since the 1980s 39, 40, 41, 42, 43, and the signs are still in clinical use 15. In particular, heart rate variability, analysed either via its frequency content 39 or various entropy measures 44, in combination with blood pressure or cardiorespiratory co‐ordination 30, 31, have been used to assess the activity of the autonomic nervous system as an indicator of the state of anaesthesia. However, our results yield insights into the changes in cardiovascular interactions revealed by the application of recently‐introduced non‐linear dynamic of analytical methods 9. They were applied, not just to one or two signals, but to several simultaneously measured signals, and a classification method was used for optimal combination of all the inferred parameters. A number of distinct changes between the awake and anaesthetised states, and between the effects of the two anaesthetics, are thus demonstrated. The study can draw two types of conclusion: (a) on which physiological parameters change significantly during anaesthesia with either propofol or sevoflurane; and (b) on which of the extracted parameters can be used to discriminate the data optimally to classify each of the subject's measured states.

Respiratory rate, skin temperature and pulse transit time increased during anaesthesia, whereas skin conductivity and the derived quantities of heart rate and respiratory rate variability both decreased. We have shown that anaesthesia is associated with a consequent reduction in the phase coherence, whereas cardiorespiratory phase synchronisation increases; the tendency for heart rate and respiratory rate to synchronise under anaesthesia is previously unreported for sevoflurane in humans. Furthermore, there was a general reduction in phase coherence between the various signals under anaesthesia and a clear difference between the effects of the two anaesthetics.

In earlier studies, heart rate variability has mostly been assessed using Fourier‐based methods of spectral analysis, which typically deal with high‐frequency (approx. 0.02–0.15 Hz) and low‐frequency (approx. 0.15–0.4 Hz) bands. While there are a number of theories for the physiological basis of the activities at different frequencies, the move from what might be termed a ‘cardiocentric’ view of the circulation to one which encompasses the role of vascular regulation is well supported by our previous work [8, 22]. Decreases have been reported in these bands 44, which are approximately equivalent to bands III and IV in our study (Table 1).

Other new findings in this study arise from the use of wavelet analysis to further refine the results of a number of physiological responses to anaesthesia. Wavelet analyses enabled us to choose window sizes according to the time scales of interest and to identify the physiological processes associated with particular frequency bands (see Table 1). We have observed for the first time a statistically significant decrease in heart rate variability wavelet en ergy in the lower frequency band V (0.0095–0.021 Hz) between the awake and anaesthetised states with both anaesthetic agents (Fig. 2a). In microvascular studies, activity in these bands has been shown to be associated with nitric oxide‐related endothelial activity 45, 46.

There was a significant increase in the mean respiratory rate with sevoflurane. Propofol resulted only in a slight increase in respiratory frequency, which was significantly lower than the average respiratory frequency during sevoflurane anaesthesia. Furthermore, the total energy of respiratory frequency variability decreased with anaesthesia, significantly with sevoflurane (p* *=* *0.02) and borderline significantly with propofol, (p* *=* *0.051). The wavelet energy of respiratory frequency variability has not previously been studied during anaesthesia.

In relation to interactions, we observed an increased tendency for the heart and respiration to synchronise during anaesthesia with both sevoflurane and propofol. This was reported in an earlier study of cardiorespiratory interactions using propofol in humans 14, and has been observed in rats 12 anaesthetised with ketamine, but has not previously been reported for sevoflurane in humans. Further, when wavelet phase coherence was used to check for significant phase relationships between the oscillations in the various signals, we observed that this relationship changed when a person is anaesthetised. For instance, between the conductivity and pulse signals, there is a reduction in the phase coherence at frequencies below 0.145 Hz for both anaesthetic agents. The observed reduction agrees with the general picture that interactions within the cardiovascular system are reduced by anaesthesia because communication through the neuronal network is restricted.

In the discriminatory classification, we studied separately the classifications based on the following parameters obtained from analysis: (a) the mean values and total wavelet energies; (b) the wavelet energy spectra for individual frequency bands; and (c) the wavelet phase coherence and synchronisation values. In each of the three categories, an optimal set was selected and the classification success rate was evaluated. Using the mean data alone, we were able to categorise subjects into three groups (awake, anaesthetised with sevoflurane, anaesthetised with propofol) with a 86% success rate, and into two groups (awake or anaesthetised) with a 90% success rate. The changes in the oscillations and their interactions induced by the anaesthetics are evidently not robust enough to provide measures of state by themselves. However, a combination of the mean values, oscillations and interaction data improved the success rate of categorising into three groups (awake, anaesthetised with sevoflurane, anaesthetised with propofol) to 90%, whereas for classifying into two groups (awake, anaesthetised) the optimisation of classification improved to 95%.

The main aims of the study were to understand the effects of the two anaesthetics and, in particular, to establish which physiological parameters change most significantly on entering anaesthesia. Thus, we hoped to identify which parameters are potentially the most useful in discriminating between the awake and anaesthetised states. These were found to be: the mean respiratory rate; the total wavelet energy of the heart rate variability; the total wavelet energy of the respiratory frequency variability signal; the mean temperature; the mean synchronisation time; the window lengths for 1:n and 2:n synchronisation; the phase coherence between pulse and temperature in frequency interval I; the wavelet energy in frequency intervals III and IV of heart rate variability; the wavelet energy in frequency interval V of the respiratory frequency variability; and the wavelet energy of frequency interval VI of the conductivity signal. Coherence and synchronisation analyses remain candidates for inclusion within a future novel method of anaesthesia monitoring, but do not provide robust enough measures for use by themselves.

Our result of 95% prediction of the awake‐to‐anaesthetised transition compares favourably with the 88% prediction based on a combination of EEG and static physiological variables 7. Once the number of data sets for classification is larger (several hundreds), one could expect prediction approaching 100%.

We have selected a relatively simple model of anaesthesia – to evaluate the effects of each of the two drugs as specifically as possible. However, to evaluate the practical applicability of the presented results, a study with more realistic conditions need to be performed. This should include: subjects with varying drug concentrations during anaesthesia, or different combination of anaesthetics; subjects who are receiving neuromuscular blocking drugs in addition to general anaesthetics; and subjects whose lungs are being mechanically ventilated, all preferably with continuous recordings during surgery and emergence from anaesthesia.

In summary, we have presented thorough investigations of the effects of sevoflorane and propofol on cardiovascular regulation. Subtle differences in the effects of these two drugs, which are known to operate via different cellular mechanisms, were identified for cardiorespiratory interactions. Furthermore, a number of relevant parameters were extracted and those that contributed to higher classification rates, in either two groups (control and anaesthetised) or three groups (control, sevoflurane and propofol) were discussed. Although we did not use measures of depth of anaesthesia other than end‐tidal sevoflurane and target propofol concentrations, we believe that our study paves the way to a modern depth of anaesthesia monitor utilising simultaneous recordings of cardiovascular signals, with on‐line non‐linear analysis and classification, able to monitor robustly the state and depth of anaesthesia. Future work could usefully validate the novel algorithm on data from a new, larger group of patients and incorporate an additional measure of depth of anaesthesia such as the use of the isolated forearm technique. We would also advocate further studies to improve the sensitivity and specificity of each of our components for the composite analyses. It has, for instance, been shown that refinement of the raw skin conductivity values into analyses of frequency of minor sweat bursts increases the precision of the skin conductivity tool 47.

## Competing interests

This work was supported by the European Union as a NEST (New and Emerging Science and Technology) Project, No. 517133, ‘Brain, Respiratory and Cardiac Causalities in Anaesthesia’ (BRACCIA http://www.physics.lancs.ac.uk/braccia), and in part by the by the Engineering and Physical Sciences Research Council (UK) (Grant No. EP/100999×/1) and by the Slovenian Research Agency (Grant No. P2‐0232). LWS was partially supported by NERC grant NE/I011889/1. There are no competing interests to declare.

## Supporting information


**Appendix S1.** Derivation of heart rate variability and respiratory frequency variability.
**Appendix S2.** Wavelet transform, wavelet phase coherence and synchronisation.
**Appendix S3.** Automatic classification.
**Table S1.** The parameters used in the vector‐based discriminatory analysis for the different subsets of data.
**Table S2.** Five confusion matrices, giving the likelihoods of correct and incorrect classification for each of the 3 groups of subjects, for five different sets of parameters.
**Figure S1.** Illustration of the vector‐based discriminatory analysis.
**Figure S2.** J48 decision tree obtained from the complete dataset.
