# Supplementary material for: The discriminatory value of cardiorespiratory interactions in distinguishing awake from anaesthetised states: a randomised observational study
Source: Anaesthesia. 2015 Sep 9;70(12):1356–68. doi: 10.1111/anae.13208 (PMC4989441; doi:10.1111/anae.13208)
Supplement: Supplementary file 1 — Appendix S1. Derivation of heart rate variability and respiratory frequency variability. Appendix S2. Wavelet transform, wavelet phase coherence and synchronisation. Appendix S3. Automatic classification. Table S1. The parameters used in the vector‐based discriminatory analysis for the different subsets of data. Table S2. Five confusion matrices, giving the likelihoods of correct and incorrect classification for each of the 3 groups of subjects, for five different sets of parameters. Figure S1. Illustration of the vector‐based discriminatory analysis. Figure S2. J48 decision tree obtained from the complete dataset. [file ANAE-70-1356-s001.doc]

**Appendix S1 Derivation of heart rate variability and respiratory frequency variability**

In order to determine the cardiac and respiratory frequency variabilities, we needed to obtain the periods of each of their cycles. The cardiac and respiratory signals can both be reduced to the position in time of the peaks in their amplitudes, each representing a defined point in the cycle of one oscillation. The ECG signal provides a distinct R-spike at every cycle which can be used as a marker for a heartbeat. Typically this is of much larger amplitude than noise from movement artefacts, making detection easily reproducible. The respiration signal has a sine-like waveform whose maxima can be used to define each breathing cycle. They represent the maximum expansions of the chest which, again, are the sections least susceptible to noise. From these, the period of each cycle can be defined as the time between two consecutive peaks. We define the instantaneous frequency as the inverse of this period, whence

$$f(t_{k})=\frac{1}{t_{k+1}-t_{k}}$$

where *t_k_* is the time of the *k*th peak. Linear interpolation between *f*(*t_k_*) values gives a frequency at every point in time. Hence we have the heart rate and respiratory frequency variabilities (HRV and RFV respectively).

Typically in clinical literature, heart rate and respiratory frequency are given in units of beats/cycles per minute. However, it is well-known that the time between consecutive R-peaks can change between each beat. As a normal healthy heart beats around once every second, we have used Hertz (cycles per second) as a measure of heart frequency consistently throughout the results.

**Appendix S2 Wavelet transform, wavelet phase coherence and synchronization**

*Wavelet transform.* Biological signals are in general governed by many rhythms, each with its own degree of influence which can change with time. They are nonlinear, in that the response to a stimulus is in general not proportional to the magnitude of the stimulus. Furthermore, their characteristic frequencies are time-varying. For instance, the heart rate is modulated by respiration (as in respiratory sinus arrhythmia), such that the heart rate changes depending on whether we are breathing in or breathing out. In order to identify the frequencies characterising a signal, we must turn to spectral analysis. Although classical spectral techniques such as Fourier analysis provide good frequency information for a signal that does not vary in time, a broadening of peaks is seen if the signal is time-variable and we cannot follow these changes in time. Wavelet analysis provides optimal *time-frequency* resolution for studying the multitude of frequencies typically observed in biological signals. The continuous wavelet transform of a signal *g(t)* recorded for a time-window *L* may be defined as


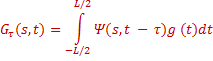


where *Ψ(s, t)* is the mother wavelet, which is scaled according to the parameter *s* to change its frequency distribution, and time-shifted according to
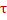
. We used a simplified complex Morlet mother wavelet [1],

$$\text{ }\psi\text{(}s_{k}\text{,}t_{n}\text{)}=s_{k}^{\text{-1/2}}\text{e}^{\text{i}t_{n}\text{/}s_{k}}\text{e}^{\text{-}t_{n}^{2}\text{/2}s_{k}^{2}},$$

where s_k=_2π/ω_k_, the index *k* denotes *k*-th frequency for which the transform is calculated and t_n_ =
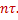
 We calculated the transform within the frequency interval 0.0095-2Hz (Table 2) and investigated the energy content within each of the five characteristic frequency intervals previously identified [2,3]. The signals we analyse are either instantaneous amplitudes (skin temperature and skin conductivity) or instantaneous frequencies. The heart and respiratory rate variabilities are in fact the heart and respiratory instantaneous rates of change of phase, or instantaneous frequencies. Thus, the energy content obtained represents the amount of variability – either in amplitude or in rate (instantaneous frequency). The complex wavelet transform$\mathop{\tilde{g}}\limits\left(s_{k},t_{n}\right)$, obtained with the Morlet mother wavelet, describes the spectral properties of the signal *g(t)* at particular points of the time-frequency domain

$\mathop{\tilde{g}}\limits\left(s_{k},t_{n}\right)=\sum_{\tau} g(t_{n}-\tau)\psi(s_{k},\tau)$.

For each time *t_n_* and scale *s_k_*, the complex transform has an amplitude *A_k,n_* and phase *φ_k,n_*:

$\mathop{\tilde{g}}\limits\left(s_{k},t_{n}\right)=A_{k,n}e^{i\varphi_{k,n}}$.

We define the total wavelet energy as$\int\left|g\left(t\right)\right|^{2}dt$; therefore the energy within each frequency interval depends on the square of the amplitude of the oscillation.

*Wavelet phase coherence and windowed wavelet phase coherence.* Because wavelet analysis with the Morlet mother wavelet may be seen as a generalisation of Fourier analysis, wavelets can also be introduced into other methods that are based on the Fourier transform and where we face the problems of time-variability and finiteness of the time series. In general, at any given point in time there is a phase difference between two oscillating signals. For non-time-variable signals (i.e. signals of constant frequency) this will be remain the same for all time. However, if the signals are time-varying then the difference in their phase relationship can change. If the phase difference tends to be maintained and to remain bounded despite the time variability, then we can infer that there is a relationship between the two signals. Wavelet analysis may show that two signals have power at the same frequency, so there is a chance that the oscillations are due to the same cause, or the oscillations have become synchronized. By using phase coherence, we can determine whether or not these oscillations are correlated [4-6].

For two signals *g*_1_(*t*) and *g*_2_(*t*), the relative phase difference
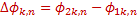
 can be computed for a given scale at each point in time. The coefficients $\cos\Delta\varphi_{k,n}$ and $\sin\Delta\varphi_{k,n}$ can be averaged in time for the whole length (*L=N*
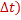
 of the time-series

$\left\langle\cos\Delta\varphi_{k,n}\right\rangle=\frac{1}{N}\sum_{n=1}^{N} \cos\Delta\varphi_{k,n},\begin{matrix} & \end{matrix}\left\langle\sin\Delta\varphi_{k,n}\right\rangle=\frac{1}{N}\sum_{n=1}^{N} \sin\Delta\varphi_{k,n}$.

The time-averaged wavelet phase coherence function is then defined as

$C_{\varphi}\left(s_{k}\right)=\sqrt{\left\langle\cos\Delta\varphi_{k,n}\right\rangle^{2}+\left\langle\sin\Delta\varphi_{k,n}\right\rangle^{2}}$.

The function $C_{\varphi}\left(s_{k}\right)$ takes values between 0 and 1. When the phase difference between the oscillations in the two time series at the frequency $\omega_{k}=\frac{2\pi}{s_{k}}$ remains constant for all time (i.e. the oscillations are coherent) then $C_{\varphi}\left(s_{k}\right)$=1. If $C_{\varphi}\left(s_{k}\right)\approx0$, there is no phase coherence between the signals *g*_1_(*t*) and *g*_2_(*t*) at the frequency. The finite length of the time-series limits the ability to average the coefficients, and so the function $C_{\varphi}\left(s_{k}\right)$ is always biased towards 1 at the longest scales, and has an uninformative low-frequency maximum. At higher (particularly cardiorespiratory) frequencies where many cycles of data are available within the period of measurement, high $C_{\varphi}\left(s_{k}\right)$ would correspond to complete coherence, and $0<C_{\varphi}\left(s_{k}\right)<1$ would correspond to partial coherence.

In order to avoid treating the frequency-dependent bias, we report only the significant changes in phase coherence in respect of phase coherence calculated from surrogate data [7]. The surrogate signals are generated by randomizing the correlations between the two signals and thus making them independent and phase incoherent, but preserving the statistical properties of each signal.

*Phase synchronization.* Phase synchronization between two oscillatory processes occurs as an adjustment of their rhythms due to interactions [8]. In such cases the phase difference between the two oscillations has constant or bounded relationship. Therefore, by quantifying how much the phase relationship is bounded or non-divergent, one can determine if there is potential phase synchronization. To do so we have used a phase synchronization index *I* based on the conditional probability of the phase relationships [9]. The method first divides each phase interval into *N* bins. Then, for each bin *l* we calculate the dependence of the instantaneous phase
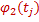
, such that phase
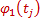
 belongs to the same bin
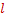
, with
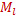
 being the number of points in the bin. The average over all bins leads to the final index:


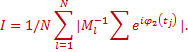


In this way, the index
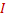
 measures the conditional probability for
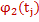
 to have a certain value provided
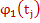
 is in a particular bin. We took *N=10* as the number of bins, with window length adjusted relative to the mean respiration period. The phase synchronization index is similar to wavelet phase coherence, but with the difference that the index
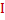
 is a time-resolved measure because it is evaluated from the instantaneous phases at each time
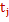
.

**Appendix S3 Automatic Classification**

***Brief overview of classification***

The primary classification technique we use is distance-based, with the simplest classification rule possible, whereby a new subject is classified to the group of its nearest neighbour from the training set. This is called the *nearest neighbour classifier*. The simplicity of the rule makes the results easily interpretable. The chosen rule is a simple extension of the other analysis performed, where we try to classify subjects based on comparing values for individual attributes.

**Figure S1: Illustration of the vector-based discriminatory analysis: each dot represents the data of a patient, with the colours representing different physiological states: awake, anaesthetised with propofol and anaesthetised with sevoflurane. By choosing an appropriate distance measure, the data points corresponding to the three different states should become separated (right).**

The distance between data vectors is given by the distance measure. We follow the idea that, with an appropriate distance metric, these vectors form well-isolated *clusters* [10] corresponding to different states, as illustrated in Fig. S1, and this is good starting point for classification. The square of the *distance measure*, denoted by *D*, is in the class of positive symmetric bilinear forms or given with the formula

*D(****x****,****y****)^2^ = (****x****-****y****)^T^* ***A*** *(****x****-****y****) ,*

where ***A*** is a symmetrical and positive matrix and ***x*** and ***y*** are data vectors with the values of attributes corresponding to the subjects. The matrix elements of ***A*** are optimised with respect to classification efficiency, given later. The distance measure in use can be seen as a merger of the plain Euclidean distance and the *feature enhancement* via linear
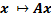
 transformation of data points, which is usually part of classification pre-processing.

The classification efficiency is measured as the probability that a subject is correctly classified, called the *accuracy* or true (positive and negative) rates. This was obtained by *repeated 50% hold-out validation*: we randomly split the data set into two halves, called the *training set* and the *testing set*, count correctly classified subjects from the testing set based on the training set and then repeat this (10,000 times) to obtain a robust statistical estimate of conditional probabilities *P(g'|g)* for the likelihoods that subjects from group *g* would fall into *g'*. If we organise the condition probabilities into a table


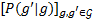
,

we get a *confusion matrix* expressed in percentages, which summarises the actual versus predicted classifications. The accuracy for classification into three groups, denoted by *A*_3_, is calculated as a weighted average of conditional probabilities to be correctly classified into each of the groups


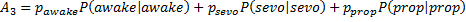
,

where *p*_awake_, *p*_sevo_ and *p*_prop_ are the percentage of the subjects corresponding to each of the three groups, and *P(awake|awake)*, *P(sevo|sevo)* and *P(prop|prop)* are the conditional probabilities of correct classification. From these results, we deduce the conditional probabilities for classification into two groups, i.e., awake and anaesthetised as explained in the next subsection. The conditional probability to correctly classify an anaesthetised subject is given by


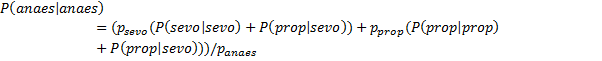


where *p*_anaes_ = *p*_sevo_+*p*_prop_. The accuracy for classification into awake and asleep groups is then calculated as


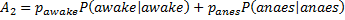
.

We restrict our efficiency analysis to the accuracy and conditional probabilities. There are many other means to quantify classification efficiency (usually applied to specific classes), such as ROC curves. Extensive reading on this topic can be found in [11].

The chosen combination of distance measure, classifier, validation and optimization, that define the classification technique, is a natural extension of statistical tests already performed on the data. To the best of our knowledge, this combination is not fully supported by currently popular classification packages, such as WEKA [12] and Orange [13]. When the classification training phase is finished, the distance measure is tailored to give the best possible performance of the classification for the chosen combination and can be applied to new data vectors with all currently available data set used as the training set.

In classification analysis, we use *two variants of the distance measures*: plain distance with a diagonal matrix ***A*** and general quadratic distance with all matrix elements of ***A*** subjected to variation. If we consider *d* attributes then the plain distance has *d* independent parameters and the general distance has *d(d+1)/2* independent parameters.

In order to keep the time complexity of accuracy optimization at a manageable level we first use plain distance on a larger number of attributes and then on only a few, usually four, most relevant attributes we apply also the general quadratic measure. We rank attributes according to their classification relevance by calculating the optimal accuracy for all possible 2^d^ subsets of attributes using plain distance and check which attributes are used in most accurate classifications.

***Primary classification method in detail***

The considered supervised classification method is based on a geometrical interpretation of the data, where we assume that subjects with similar medical data are in the same medical state, forming a cluster when given an appropriate metric. We determine which data points are nearer to each other by applying a measure of distance on the sets of measurements.

Let us consider a general case of *n* subjects in *m* groups labeled by integers. The *i*th subject is characterized by a vector of attribute values
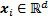
and associated to some group
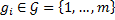
, where we assume that the data is unique:
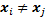
 for
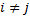
. For convenience we introduce the set of all data vectors


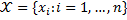


and the set containing all information about the subjects


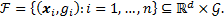


where pairs
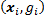
 is referred as instances. In order to simplify the association of the data vectors to the groups, we introduce the function
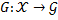
 that assigns to each vector in
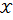
 its corresponding group based on information in
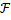
:


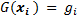
 for all *i*.

The relative size of the *i*th group can then be written as

,

and the relatives sizes sum up to 1:
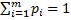
. For given data vectors ***x*** and ***y***, the distance measure *D(****x****,****y****)* is defined as


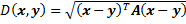
,

where ***A=L^T^L*** is a symmetric and positive matrix representing the metric tensor and is expressed via a lower triangle matrix ***L***. We use two variants of the distance measure: plain distance with diagonal matrix ***L***, and consequently matrix ***A***, and general quadratic distance where all elements of ***L*** are mutable.

We define the classification as a prognosis based on some prior knowledge. Taking a subset of data
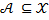
, called the training set, then the classification function
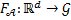
 associates to data vector
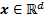
 group
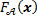
 corresponding to a member of
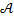
 nearest to
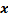
:


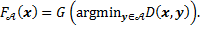


Note that
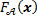
 is invariant on the scaling of distance parameters, i.e., rescaling of the metric tensor
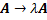
 yields the same result. We check the success of classification by *50% hold-out validation*, i.e., splitting the data set
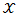
 into learning set
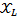
 and testing set
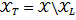
 of approximately the same size and counting how many elements of
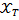
 that can be identified to be in group
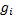
are associated/classified to group
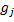
 using the classifier
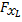
, which is judging based on knowledge
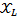


.

Correctly associated subjects are accounted for in the diagonal
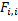
. Instead of absolute numbers, we rather discuss empirical probabilities that a subject in group
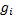
is classified to group
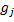
 given by


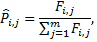


for a pair of sets
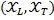
. In order to quantify the general success of the classification using some distance measure
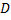
 we average empirical probabilities over all possible pairs of set
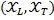
 with size of
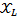
 being approximately half of the size of
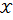
 and obtain
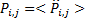
. For a given database, distance measure *D* and used type of validation the *confusion matrix* (or more precisely its probability variant) is given by


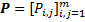


For the classification as a Markov process, the matrix element
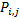
 represent a conditional probability *P(j|i)* that a subject/instance from group
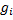
 is classified into group
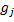
:a randomly peeked subject from group *­*
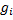
 is classified into group
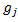
 with probability
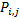
 or correctly classified with probability
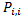
.The general success of classification is given by the average probability of correctly classifying a subject/instance


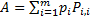
 ,

referred to as *classification accuracy.* This is the measure that we maximize by varying the parameters of the distance measure in the matrix ***A*** and with that, specialize the distance measure for classification with the given database of patients. We optimize the classification accuracy by first finding a vicinity of a global maximum via a greedy Monte Carlo by checking N=10^6^ cases of distance parameters from a meaningfully selected bounded set. Next, we further optimize the accuracy, and polish distance parameters, with the Nelder-Mead method [14] in order to reach its local or even possibly a global maximum.

The classification can be performed using different number of attributes. Theoretically speaking, larger is the set of attributes, better you can describe a medical state of a subject. In practice the situation is more complicated, as the measured values of attributes have errors which can obstruct a correct classification. In the following we investigate how the classification performs on different subspaces of *d* attributes. Let use a binary code
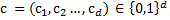
to mark which attributes are used classification:


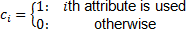
.

Certain subsets of attributes are better for classification than the others and this is indicated by higher corresponding optimal accuracy *A*. We quantify the relevance of individual attributes for (the high accuracy of) classification by introducing a *measure of relevance:* for the *i*th attribute this measure reads


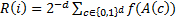
,

where *f* is a monotonically increasing cost function and
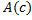
 is the accuracy corresponding to binary code *c*. This is a very costly operation and can be done to satisfactory precision only for a smaller *d*. To get visually interpretable results for the relevance of attributes we use
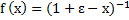
 with the machine precision *ε > 0* to avoid possible numerical singularities. By sorting the
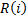
 we can rank attributes for efficiency of classification. Due to large time complexity
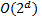
this method is only applicable, when the number of parameters *d* is small. To the best of our knowledge this way of quantifying relevance of attributes for classification is our original contribution or at least is not widely used.

***Classification Results***

It would have been computationally too time-consuming to classify on the basis of all parameters simultaneously. Classifications were therefore considered separately for each parameter set obtained through the analyses described above: (a) mean values; (b) parameters characterising oscillations from the wavelet analysis; (c) parameters characterising their interactions from the phase coherences and synchronization. Next, the classification method was applied to the optimal parameters from (b) and (c). Finally, classification was applied to the optimal parameters obtained from (a)-(c).

*Mean values data set (a)*. We initially worked with12 attributes shown in Table S1, column 1. The maximal achieved average probabilities of correct classification, i.e. classification accuracy denoted by *A*, using the plain distance were *A*_3_ = 86% and *A*_2_ = 90%. By optimizing the general distance on the four most relevant attributes for the plain distance (shaded in Table S1, column 1) the maximal average probabilities became *A*_3_ = 84% and *A*_2_ = 89%, whereas using the general quadratic distance we obtain *A*_3_ = 88% and *A*_2_ = 91%. Full details appear in Table S2 (A).

*Wavelet energy data set (b)*. Optimization of the plain distance using 15 attributes (Table S1, column 2) yielded maximal average probabilities of *A*_3_ = 75% and *A*_2_ = 88%. Again, by reducing the space to the four most relevant attributes for plain distance (shaded in Table S1, column 2), the probabilities were *A*_3_ = 75% and *A*_2_ = 84%, whereas with the general quadratic distance this improved to *A*_3_ = 80% and *A*_2_ = 86%. Full details appear in Table S2 (B).

*Interactions data set (c).* Initially we have used 9 attributes listed in Table S1, column 3. This yielded the maximal average probabilities of *A*_3_ = 75% and *A*_2_ = 88%. By optimizing to the four most relevant attributes (shaded in Table S1, column 3), the probabilities for correct classification using plain distance were *A*_3_ = 76% and *A*_2_ = 82%. Using the general quadratic distances this improved to *A*_3_ = 79% and A_2_ = 84%. Full details appear in Table A2 (C).

Combining the discriminatory value of the most relevant un-normalised wavelet energies and interactions values (Table S1, column 4) i.e. working on an 8-dimensional space, we obtain *A*_3_ = 84% and *A*_2_ = 89%. For the four most relevant dimensions for plain distance and optimizing general distance on that space, we obtain *A*_3_ = 82% and *A*_2_ = 87%. Using the general quadratic distances we obtain correct classification probabilities of *A*_3_ = 85% and *A*_2_ = 87%. Full details appear in Table S2 (D).

Finally, we tested the discrimination based on the four most relevant attributes from each of the three data sets - mean values, un-normalised wavelet energies and interactions values - taken together, i.e., working on 12 attributes (Table S1, column 5). This achieved further improvement to probabilities of *A_3_* = 90% and A_2_ = 95%. For the four most relevant dimensions (shaded in Table S1, column 5) by using plain distance we had *A*_3_ = 86% and *A*_2_ = 89%. The general quadratic distance yielded discriminatory probabilities of *A_3_* = 90% and *A_2_* = 92%. Full details appear in Table S2 (E).

**Table S1** The parameters used in the vector-based discriminatory analysis for the different subsets of data. Roman numerals indicate frequency intervals (see Table 2 in main paper). Where the subsets have subsequently been reduced, the four attributes are shaded.

| *Mean values subset* | *Wavelet powers subset* | *Interactions subset* | *Wavelet powers and interactions combined subset* | *Mean values, wavelet powers and interactions combined subset* |
| --- | --- | --- | --- | --- |
| Heart rate | HRV energy II | HRV-conductivity II | HRV energy II | Respiratory rate |
| Respiratory rate | HRV energy III | HRV-conductivity III | HRV energy IV | Total HRV energy |
| Skin temperature | HRV energy IV | HRV-conductivity IV | RFV energy III | Total RFV energy |
| Skin conductivity | HRV energy V | Conductivity-pulse I | RFV energy IV | Total temperature energy |
| Pulse transit time | RFV energy III | Conductivity-pulse III | Pulse-temperature I | HRV energy II |
| Total HRV energy | RFV energy IV | Conductivity-pulse IV | C-R synchronization time | HRV energy IV |
| Total RFV energy | RFV energy V | Conductivity-temperature I | 1:n synchronization window length | RFV energy III |
| Total conductivity energy | Conductivity energy III | Conductivity-temperature II | 2:n synchronization window length | RFV energy IV |
| Total temperature energy | Conductivity energy IV | Pulse-temperature I |  | Pulse-temperature I |
|  | Conductivity energy V | C-R synchronization time |  | C-R synchronization time |
|  | Conductivity energy VI | 1:n synchronization window length |  | 1:n synchronization window length |
|  | Temperature energy III | 2:n synchronization window length |  | 2:n synchronization window length |
|  | Temperature energy IV |  |  |  |
|  | Temperature energy V |  |  |  |
|  | Temperature energy VI |  |  |  |

**Table S2** Five confusion matrices**,** giving the likelihoods of correct and incorrect classification for each of the 3 groups of subjects, for five different sets of parameters: A) the mean values data set, B) the wavelet power data set, C) the interactions data set, D) the most relevant wavelet powers and interactions data set and E) the four most relevant mean values, four most relevant wavelet powers and four most relevant interactions values. The likelihoods of correct classification, for each of the 3 groups of subjects, are shown in bold.

|  |  | Classification outcome | | |
| --- | --- | --- | --- | --- |
| A. Mean values data set | | *Awake* | *Anaes-Sevoflurane* | *Anaes-Propofol* |
| Actual  state | *Awake* | **92%** | 2% | 6% |
|  | *Anaes-Sevoflurane* | 7% | **84%** | 9% |
|  | *Anaes-Propofol* | 19% | 7% | **74%** |
|  |  |  | | |
| B. Wavelet powers data set | |  |  |  |
| Actual  state | *Awake* | **89%** | 2% | 9% |
|  | *Anaes-Sevoflurane* | 15% | **72%** | 13% |
|  | *Anaes-Propofol* | 11% | 42% | **47%** |
|  |  |  | | |
| C. Interactions data set | |  |  |  |
| Actual  state | *Awake* | **85%** | 5% | 10% |
|  | *Anaes-Sevoflurane* | 21% | **63%** | 16% |
|  | *Anaes-Propofol* | 39% | 31% | **30%** |
|  |  |  | | |
| D. Most relevant wavelet powers and interactions combined data set | |  |  |  |
| Actual  state | *Awake* | **90%** | 2% | 8% |
|  | *Anaes-Sevoflurane* | 7% | **88%** | 5% |
|  | *Anaes-Propofol* | 19% | 14% | **67%** |
|  |  |  | | |
| E. Most relevant mean values, wavelet powers and interactions combined data set | |  |  |  |
| Actual  state | *Awake* | **94%** | 2% | 4% |
|  | *Anaes-Sevoflurane* | 7% | **84%** | 9% |
|  | *Anaes-Propofol* | 12% | 7% | **81%** |

***Other standard classification techniques and associated analysis***

We also used several standard classification techniques from the software package Waikato Environment for Knowledge Analysis (WEKA) [12]. We compared the following classification (predictive modeling) techniques: decision trees (J48 in WEKA), classification rules (JRip), decision tree ensembles (random forests, bagging), support vector machines (SMO), neural networks (MLP). These were applied on the whole set of attributes and their accuracy was estimated by leave-one-out (50-fold) cross-validation. We found that the most successful method was the *J48 decision tree/C4.5 algorithm* *release 8* (WEKA class weka.classifiers.trees.J48): The decision tree presented in Fig. S2 had an estimated accuracy of 78% (on unseen cases).


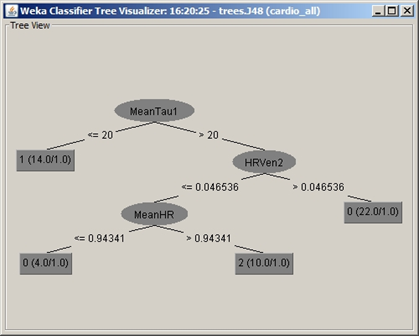


|  |
| --- |
| **Figure S2**: J48 decision tree obtained from the complete dataset. The internal nodes (of oval shape) are labelled with names of attributes they examine, the branches leading from internal nodes downwards are labelled with tests on attribute vales, and leaf nodes (of rectangular shapes) are labelled with indices of target groups (0 – awake, 1 anaesthetised with sevoflurane, 2- anaesthetised with propofol). The numbers in parenthesis in a leaf node denote the total/misclassified number of instances that reach the leaf. |

The confusion matrix, giving the likelihoods of correct and incorrect classification for each of the 3 groups, resulting from the leave-one-out (50-fold) cross-validation, is given below

| Cardio – D. trees - J48 | | Awake | Anaes-Sevoflurane | Anaes-Propofol |
| --- | --- | --- | --- | --- |
| Actual  State | Awake | 84% | 4% | 12% |
|  | Anaes-Sevoflurane | 7% | 86% | 7% |
|  | Anaes-Propofol | 45% | 0% | 55% |

The WEKA package also includes different approaches for estimating the relevance of attributes for successful classification and description of groups. One such approach is the Recursive Elimination of Features-F (acronym RELIEF-F, WEKA class weka.attributeSelection.ReliefFAttributeEval), which calculates for each attribute a merit value estimating the relevance of the attribute for describing the target groups. This merit value takes into account the interaction of the attribute with other attributes [15]. By sorting RELIEF-F merits from highest to lowest we obtain a ranking table; here we show the top ten highest ranking attributes:

| **Average merit** | **Average rank** | **Attribute** |
| --- | --- | --- |
| 0.159 +- 0.014 | 1 +- 0 | MeanRR |
| 0.103 +- 0.015 | 2.7 +- 0.78 | MeanTau2 |
| 0.105 +- 0.015 | 3 +- 0.89 | TotalHRVen |
| 0.1 +- 0.015 | 3.9 +- 0.94 | MeanTau1 |
| 0.073 +- 0.012 | 5.9 +- 1.3 | HRVen4 |
| 0.064 +- 0.011 | 6.6 +- 1.36 | HRVen3 |
| 0.059 +- 0.016 | 8.3 +- 3.2 | MeanSynch |
| 0.051 +- 0.008 | 9.5 +- 2.42 | MeanTemp |
| 0.051 +- 0.011 | 9.8 +- 2.82 | RFVen4 |
| 0.054 +- 0.024 | 10 +- 3.55 | CohPulseTemp1 |

An alternative way to estimating relevance is to calculate the information gain of an attribute, measuring how much information about the class (in terms of entropy reduction) is contained in the attribute (method Infogain, WEKA class weka.attributeSelection.InfoGainAttributeEval). Infogain considers the contribution of each attribute individually and does not take into account its interactions with other attributes. We rank the attributes by sorting the information gain merit from highest to lowest and show the ten most relevant attributes in the table below.

| **Average merit** | **Average rank** | **Attribute** |
| --- | --- | --- |
| 0.636 +- 0.048 | 1.7 +- 0.46 | MeanTau2 |
| 0.636 +- 0.049 | 1.8 +- 0.98 | MeanTau1 |
| 0.544 +- 0.048 | 3.9 +- 1.37 | MeanRR |
| 0.485 +- 0.034 | 4.9 +- 0.94 | HRVen2 |
| 0.475 +- 0.042 | 5.1 +- 1.3 | RFVen3 |
| 0.438 +- 0.052 | 6 +- 1.55 | HRVen4 |
| 0.457 +- 0.141 | 7 +- 3.22 | RFVen4 |
| 0.414 +- 0.092 | 8.2 +- 2.64 | TotalHRVen |
| 0.346 +- 0.025 | 10.6 +- 1.2 | CondEn4 |
| 0.35 +- 0.059 | 10.8 +- 3.16 | CohPulseTemp1 |

**References**

1 Grossmann A, Morlet J. Decomposition of Hardy Functions into Square Integrable Wavelets of Constant Shape. *SIAM Journal on Mathematical Analysis*. 1984; **15**: 723–36.

2 Shiogai Y, Stefanovska A, McClintock PVE. Nonlinear dynamics of cardiovascular ageing. *Physics Reports*. Elsevier B.V.; 2010; **488**: 51–110.

3 Lotrič MB, Stefanovska A, Stajer D, Urbancic-Rovan V. Spectral components of heart rate variability determined by wavelet analysis. *Physiological Measurement*. 2000; **21**: 441–57.

4 Bandrivskyy A, Bernjak A, McClintock P, Stefanovska A. Wavelet Phase Coherence Analysis: Application to Skin Temperature and Blood Flow. *Cardiovascular Engineering*. Kluwer Academic Publishers-Plenum Publishers; 2004; **4**: 89–93.

5 Sheppard LW, Stefanovska A, McClintock PVE. Testing for time-localized coherence in bivariate data. *Physical Review E*. 2012; **85**: 046205.

6 Clemson PT, Stefanovska A. Discerning non-autonomous dynamics.

*Physics Reports* 2014; **542:** 297-368.

7 Schreiber T, Schmitz A. Surrogate time series. *Physica D: Nonlinear Phenomena* 2000;**142**:346-382

8 Pikovsky A, Rosenblum M, Kurths J. *Synchronization: A Universal Concept in Nonlinear Sciences.* Cambridge University Press, 2001.

9 Kenwright DA, Bahraminasab A, Stefanovska A, McClintock PVE. The effect of low-frequency oscillations on cardio-respiratory synchronization: Observations during rest and exercise. *The European Physical Journal B*. 2008; **65**: 425–33.

10 Jain AK, Murty MN, Flynn PJ. Data clustering: a review. *ACM Computing Surveys*. 1999; **31:** 264–323.

11 Zou KH, Liu A, Bandos AI, Ohno-Machado L, Rockette HE. *Statistical Evaluation of Diagnostic Performance: Topics in ROC Analysis.* Boca Raton: CRC Press; 2012.

12 Hall M, Frank E, Holmes G, Pfahringer B, Reutemann P, Witten I. The WEKA Data Mining Software: An Update. *SIGKDD Explorations*. 2009; **11**: 10–18.

13 Demšar J, Curk T, Erjavec A, et al. Orange: Data Mining Toolbox in Python. *Journal of Machine Learning Research*. 2013; **14**: 2349–53.

14 Press WH, Teukolsky SA, Vetterling WT, Flannery BP. *Numerical recipes in C: the art of scientific computing.* New York:Cambridge University Press, 1988

15 Robnik-Sikonja M, Kononenko I. Theoretical and Empirical Analysis of ReliefF and RReliefF. *Machine Learning Journal* 2003; **53:**23-69.
